# Primary Angiitis of the Central Nervous System Mimicking Glioblastoma: A Case Report and Literature Review

**DOI:** 10.3389/fneur.2019.01208

**Published:** 2019-11-13

**Authors:** Hang Jin, Yang Qu, Zhen-Ni Guo, Guo-Zhen Cui, Fu-Liang Zhang, Yi Yang

**Affiliations:** ^1^Department of Neurology, Stroke Center, The First Hospital of Jilin University, Changchun, China; ^2^Department of Neurology, Clinical Trial and Research Center for Stroke, The First Hospital of Jilin University, Changchun, China; ^3^Department of Hepatology, Cancer Center, The First Hospital of Jilin University, Changchun, China

**Keywords:** primary angiitis of the central nervous system, tumor-like lesion, vasculitis, imaging diagnosis, malignant glioma

## Abstract

Primary angiitis of the central nervous system (PACNS) is a rare disorder resulting in idiopathic inflammation affecting the parenchymal and leptomeningeal vessels confined to the central nervous system (CNS), of which a tumor-like mass lesion is an even rare subtype. We described a case of PACNS initially misdiagnosed as glioblastoma. The patient was a 35 year-old female with right-sided weakness and expressive dysphasia. Brain MRI showed a tumor-like lesion highly suggestive of glioblastoma, therefor surgical removal was done. After a resection and an exhaustive workup, PACNS was ultimately diagnosed. The case illustrates a type of imaging presentation of PACNS that is often misdiagnosed as high-grade glioma. Differentiation between tumor-like PACNS lesions and actual CNS tumors is challenging due to similar MR images. To avoid unnecessary surgical interventions, we summarized previously reported mass-forming PACNS cases in adults from January 1, 2000, to December 31, 2018 and the imaging characteristics of PACNS. Some less commonly used diagnostic methods such as MR spectroscopy may also help clinicians distinguish PACNS from its mimics.

## Background

Tumor-like mass lesion is a rare presentation of PACNS, accounting for only 5% of all PACNS. Accordingly, it is difficult to differentiate tumor-like PACNS from neoplastic diseases. This report describes a patient presenting with a cerebral mass lesion in the left parietal lobe, clinically consistent on initial neuroimaging with high-grade glioma, which was ultimately diagnosed of PACNS by histopathology. PACNS cases with tumor-like lesion similar to the current patient are rare. This report summarizes these cases from January 1, 2000, to December 31, 2018. Additionally, to avoid unnecessary surgical interventions, this report summarizes the imaging characteristics of tumor-like mass lesion in PACNS and some less commonly used diagnostic methods such as MR spectroscopy that may also help clinicians distinguish PACNS from its mimics.

## Case Presentation

A 35 year-old Chinese woman was admitted to our hospital 2 days after an acute onset of right-sided weakness and expressive dysphasia. She had no history of infection and vaccination within 6 weeks and no other medical history. Her family had no hereditary diseases. The patient was a salesclerk in a bookstore. She never drank alcohol or smoked. A systemic examination was unremarkable. A neurological examination revealed motor aphasia, facial asymmetry, 2/5 strength in the right upper and lower extremities, and a positive right-sided Babinski reflex.

**Figure 1:**
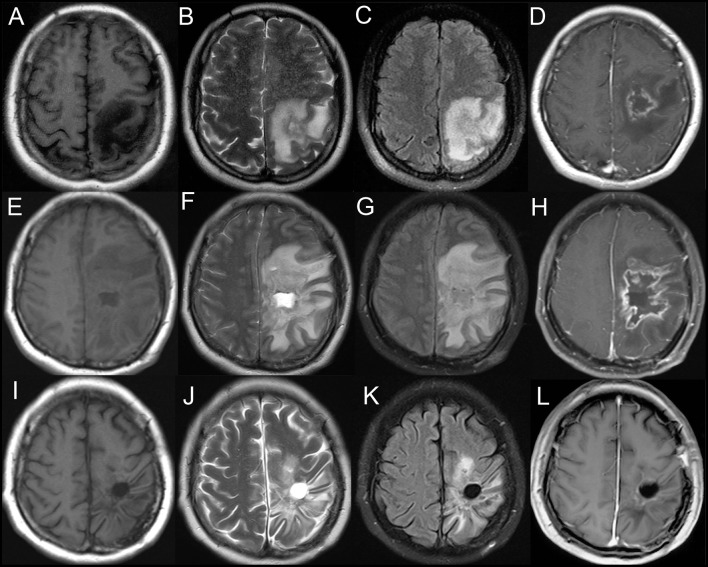
Axial T1-weighted **(A)**, T2-weighted **(B)**, and fluid-attenuated inversion recovery (FLAIR) **(C)** demonstrating a heterogeneous mass centered in the left parietal lobe. T1-weighted gadolinium-enhanced **(D)** MRI showing an irregular peripheral enhancement mass with a central non-enhancing area. **(E–H)** Postoperative MRI scan showing a flaky edema area in the left frontotemporal lobe. Axial T1-weighted **(I)**, T2-weighted **(J)**, fluid-attenuated inversion recovery (FLAIR) **(K)**, and T1-weighted gadolinium-enhanced **(L)** MRI showing no evidence of contrast enhancement in the left parietal lobe on 6 month follow-up.

**Figure 2:**
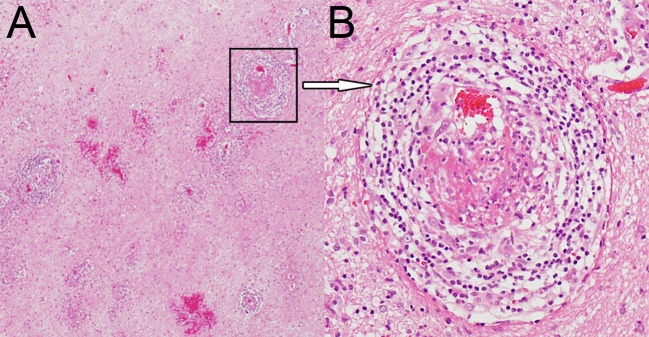
Hematoxylin and eosin staining magnified 40× **(A)** and 200× **(B)** showing dense perivascular lymphohistiocytic infiltration.

A brain MRI showed a tumor-like lesion with surrounding edema in subcortical of the left parietal lobe highly suggestive of glioma. The mass lesion demonstrated hypodensity on the brain MRI T1 image, hyperintensity on the MRI T2 image, and hypointensity on the fluid-attenuated inversion recovery (FLAIR) sequences. A gadolinium-enhanced MRI showed a solitary 2.8 cm × 2.6 cm × 4.1 cm irregular peripheral enhancement mass with a central non-enhancing area centered in the left parietal lobe (Figures 1A–D). Magnetic resonance angiography displayed mildly abnormal with no sign of vasculitis; slight narrowing was observed in the bilateral siphon carotid arteries, bilateral anterior cerebral arteries, and the M1 segment of the left middle cerebral artery. All of the preoperative laboratory findings were within the normal limits: white blood cells were 8.76 × 10^9^/L (normal, 4.0–10.0), red blood cells were 3.93 × 10^12^/L (normal, 3.5–5.1), hemoglobin was 116 g/L (normal, 115–150), and hematocrit was 0.361 (normal, 0.350–0.450). Electrolytes with glucose level, liver function test, arterial blood gas, renal function test, and thyroid function test were within the normal limits. The venereal disease research laboratory (VDRL) test was non-reactive. High-grade glioma was provisionally diagnosed and the patient underwent a mass resection. However, the histopathological examinations revealed a lymphocytic inflammatory infiltrate in the vessel walls and perivascular zones, and parts of the vessels demonstrated necrotic wall damage and contained hyaline microthrombi, consistent with central nervous system vasculitis (Figure 2). Immunohistochemical examinations showed negative staining for Ki-67, P53, GFAP, IDH1, R132H, 01igo-2, MBP, Map2, NeuN, Syn, and CD68. After surgery, the patient's consciousness deteriorated, and a postoperative MRI scan demonstrated flaky extensive perifocal edema that could not be explained by glioma (Figures 1E–H).

Once vasculitis was diagnosed, an evaluation of its secondary causes was pursued. Acute phase reactants, for example, the erythrocyte sedimentation rate (ESR) and C-reactive protein (CRP), and a complete blood count (CBC) were normal. Serum antineutrophil cytoplasmic antibody (ANCA), antinuclear antibodies (ANAs), anticardiolipin antibody (ACA), antidouble-stranded DNA, rheumatoid factor (RF), cyclic citrullinated peptide (CCP), complement 3 and 4, immunoglobulin G (IgG), IgM, and IgA remained within the normal ranges. Whole-body fluorodeoxyglucose-PET (FDG-PET) and several serum tumor markers (CA724, CYFRA21-1, CEA, CA242, CA125, NSE, AFP, SCC, CA199, CA153, HCG) suggested no evidence of metastatic tumors. Lumbar puncture showed the patient's intracranial pressure was 390 cm H_2_O (normal range, 80–180) and a cerebrospinal fluid analysis identified lymphocytic predominant pleocytosis (64 × 10^6^ cells/L, local reference range, 0–8 × 10^6^), elevated protein (0.82 g/L, local reference range, 0.15–0.45), and normal glucose levels (3.40 mmol/L, normal range, 2.3–4.1). Other CSF findings included IgG (39.00 mg/L, normal range, 0–34), IgG index (3.63, normal <0.85), IgG synthesis (151.2 mg/24 h, normal <7.0), and oligoclonal bands (positive, normal negative). The results of cytologic studies and bacterial and viral cultures were all negative. Additional testing of the CSF and serum including antibodies in various paraneoplastic neurological syndromes, myelin basic protein (MBP), MBP antibody (MBP-Ab), myelin oligodendrocyte glycoprotein antibody (MOG-Ab), and aquaporin 4 antibody (AQP4-Ab) were all within the normal ranges. Further examinations revealed no evidence of metabolic, autoimmune, or infectious disorders, systemic vasculitis, or neoplastic malignancy. PACNS was diagnosed.

The patient was commenced on intravenous methylprednisolone 1 g daily, halved every 3 days and tapered over 12 days to prednisone 60 mg daily via oral administration with 2.5 mg/week reduction, and the treatment was maintained for approximately 6 months. A repeat brain MRI after 6 months of treatment showed that the lesion had nearly disappeared with the resolution of edema and mass effect (Figures 1I–L). At a 1 year follow-up, the patient could walk independently.

## Discussion

Many inflammatory and infectious processes affect the central nervous system (CNS) and can simulate other neurosurgical pathologies (1). This report describes a peculiar case of histopathologically proven PACNS presenting a tumor-like lesion, which was initially misdiagnosed as malignant glioma, and surgical removal was done unnecessarily. This study also summarizes the imaging characteristics of tumor-like mass lesion PACNS to possibly avoid unnecessary surgical interventions.

PACNS, also known as primary central nervous system vasculitis (PCNSV), is a rare disorder resulting in idiopathic inflammation affecting the parenchymal and leptomeningeal vessels confined to the CNS (2). Secondary causes of vasculitis should be ruled out via thorough clinical and laboratory examinations (3). This disorder was first considered a distinct clinical entity in 1959 by Cravioto and Feigin (4). In 1988, Calabrese and Mallek systematically reported eight cases of this disease, named it PACNS, and proposed the preliminary clinical criteria for diagnosis (3). Supplementary clinical diagnoses were proposed by Birnbaum and Hellmann (5). In 2011, Hajj-Ali et al. proposed classification criteria based on clinical manifestations, imaging results, and pathological biopsy findings in brain tissue (6).

PACNS remains a rare disorder. A retrospective analysis of 163 cases from the Mayo Clinic (Rochester, MN, USA) over a 29 year period from 1983 to 2011 revealed that the average annual incidence rate of PACNS was 2.4 cases per 1 million person-years (7). The disease was more common in males. In the Mayo Clinic sample, the male-female ratio was ~0.8 to 1 (7). PACNS may occur at almost any age. Approximately 50% of patients are between 40 and 60 years old. The condition is very uncommon in children (8).

Major symptoms of PACNS at diagnosis are highly variable and non-specific, including headache (60%), altered cognition (50%), and focal neurologic deficits or stroke (for example, hemiparesis, ataxia, aphasia, dysarthria, and visual disturbances) (9, 10). These manifestations are also the initial symptoms in two-thirds or more of cases. The onset may be abrupt but is usually gradual and progressive (8). Less common presenting symptoms include seizures, ataxia, coma, and cranial neuropathies (11).

The results of serum and CSF tests are usually normal, which mainly helps to exclude other possible causes of the symptoms (7). A minority of PACNS patients may have mildly elevated acute-phase reactants such as ESR and CPR (7, 12–14). A previous study reported that the clinical findings and outcomes in patients with elevated ESR did not differ from those with normal ESR (7). If other serological findings including ANCA, ANAs, ACA, antidouble-stranded DNA, RF, and complement 3 and 4 are obviously abnormal, secondary factors of vasculitis should be considered. CSF findings are necessary but are generally mild and non-specific. Changes typically consisted of lymphocytic pleocytosis, increased total protein concentrations, and normal glucose levels, parallel with the results of aseptic meningitis, and occasionally with the presence of oligoclonal bands and elevated IgG synthesis (7, 15). Infectious disorder, systemic vasculitides, and neoplastic malignancy were excluded in our patient according to her serum and CSF tests, but she was also part of a small group with positive oligoclonal bands and elevated IgG synthesis.

PACNS imaging methods can be divided into three categories based on the structures examined (16), of which MRI is the most common choice in practice (17). MRI findings of PACNS are sensitive but non-specific, and several studies have reported an MRI sensitivity ~90–100% (7, 14). A wide variety of brain MRI changes occur in PACNS, and 10 of the most common have been listed (18). Most PACNS symptoms present as multiple bilateral asymmetrical segmental inflammation of the small and medium vessels of the CNS (19). However, only 5% of PACNS cases present tumor-like mass lesions (2). One of the mechanisms of the formation of a mass lesion is a breakdown of the blood-brain barrier of the small vessels via the infiltration of inflammatory cells in the perivascular and parenchymal regions, resulting in a mass-mimicking enhanced lesion (20). Amyloid angiopathy may also play a part (21). In the present case, the postsurgical flaky extensive perifocal edema was thought to be associated with vascular and perivascular inflammatory changes.

The solitary tumor-like mass lesions of PACNS are rare, which usually show high signal intensity on T2 and heterogeneously enhancing signals on Gd-enhanced images (17, 22–26). Previously reported mass-forming PACNS cases in adults from 2000 to the present are summarized in the Table 1. Most PACNS lesions occur in the supratentorial subcortical and deep white matter similar to the present patient. However, Zhang et al. (20) reported that the first case of PACNS occurring in the cerebellum was initially misdiagnosed as a malignant glioma, and the mass was unnecessarily resected via a retrosigmoid approach. Lee et al. (26) reported another case that manifested as a suprasellar mass, the preoperative diagnosis of which was a malignant tumor. Another case of PACNS that mimicked a malignant brainstem neoplasm disseminating into the ventricles and cervical spinal cord was reported in 2008, unfortunately, the patient died of respiratory dysfunction 2 weeks later (31).

**Table 1:** Summary of PACNS with a tumor-like lesion in adult patients from January 1, 2000, to December 31, 2018.

**References**	**Age at diagnosis (years) and gender**	**Clinical presentation**	**Laboratory findings**	**CSF analysis**	**Type of vasculitis**	**MRI brain results**	**Location**	**Treatment**	**Outcome**
Molloy et al. (2)	43F	Reduced coordination and visuospatial disorientation with right inferior quadrantanopia	Not mentioned	Not mentioned	Lymphocytic	A large contrast enhancing mass with edema and local mass effect	The left parietal lobe	Surgical removal, glucocorticoids, and mycophenolate mofetil	No recurrence on 21 month follow up
Lee et al. (26)	24M	Seizure	Not mentioned	Not mentioned	Lymphocytic	High signal on T2, low signal on T1, increased ADC, normal angiogram	The left frontal lobe	Corticosteroids and lesionectomy	Disappeared multiple-enhancing lesions on 8.1 year follow up
Lee et al. (26)	37F	Sleeping tendency, poor oral intake, and decreased verbal output	Not mentioned	Not mentioned	Lymphocytic	2.3 cm enhancing mass with edema, decreased ADC, MCA M1 occlusion, and left distal ICA stenosis on angiogram	Suprasellar area	Cyclophosphamide and corticosteroids	Decreased size on 1 year follow up
You et al. (27)	35F	Headache and left-sided weakness	Normal	Increased total protein: 170 mg/dl; MBP 2.23 nmol/l; increased IgG in CSF index 20.5 mg/dl	Lymphocytic	A tumor-like mass with edema and enhancement	The right parietal lobe	Subtotal resection, prednisolone, and cyclophosphamide	Neurological symptoms disappeared on 6 week follow up
Tanei et al. (24)	60M	Slight disorientation, left hemiparesis, and motor aphasia	Normal	Normal	Granulomatous	A slightly enhanced mass lesion with surrounding edema	The right parietal lobe	Surgical removal only	Improvement with no new lesions on 6 month follow up
Muccio et al. (25)	46F	Progressive confusion, headache, dizziness, and nystagmus	Not mentioned	Not mentioned	Lymphocytic	Increased signal on FLAIR, low signal on T1, and signal intensification with contrast	The right temporal lobe	Steroids	Regression of the lesion on 3 month follow up
Pizzanelli et al. (28)	50F	Speech disorder, headache, and apathy	Not mentioned	Not mentioned	Lymphocytic	Mixed hyperintensity on T2 with conspicuous edema	The left frontal lobe	Surgical removal, steroids, and cyclophosphamide	Recurrence after one year and 5 months
Killeen et al. (29)	51M	Headache and left homonymous hemianopia	Normal	Mononuclear pleocytosis: 58/L elevated protein: 1400 mg/L	Lymphohistiocytic	Extensive perifocal edema, ring-enhancing and central necrotic mass on MRI	The white matter adjacent to the posterior horn of the right lateral ventricle	Oral prednisolone and cyclophosphamide	Suffered no recurrence on 4 year follow up
Kim et al. (22)	20F	Right facial focal motor seizures	Elevated CRP: 9.80 mg/dL	Not mentioned	Lymphocytic	High signal intensity on T2 and heterogeneous enhancement with contrast	The left posterior frontal area	Surgical removal	No new lesions on 3 year follow up
Sun et al. (30)	42M	Headache, convulsions, and aphasia	Not mentioned	Normal	Lymphocytic	A tumor-like mass with edema and high signal intensity on FLAIR	The left frontotemporal lobe	Surgical removal, methylprednisolone, and subsequent oral prednisolone	Only aphasia on 1 year follow up
Zhu et al. (17)	22M	Jacksonian epilepsy and weakness in right thumb	Elevated CRP: 10.52 mg/L; elevated ESR: 22 mm/h; ANCA, ANA, and RF (-)	Elevated protein: 0.85g/L	Lymphocytic	Hypodensity on T1, hyperintensity on T2, no enhanced signal on Gd-enhanced image, and mild hyperintensity on FLAIR	The left temporal lobe	Methylprednisolone and subsequent oral prednisone	Most symptoms were relieved on 50 day follow up
Zhu et al. (17)	31M	Recurrent unconsciousness, right limb convulsions, aconuresis or encopresis, headache, and nausea	Elevated CRP: 11.78 mg/L; elevated ESR: 18 mm/h ANCA, ANA, and RF (–)	Slightly elevated protein: 0.48 g/L	Lymphocytic	Mixed hyperintensity on T1 and T2, hyperintensity on FLAIR, and DWI and Gd-enhanced images	The left temporal lobe	Methylprednisolone and subsequent oral prednisone	Only mild memory impairment remained without adverse events on 7 month follow up
Zhang et al. (20)	35F	Severe headache and gait disturbances	Not mentioned	Not mentioned	Lymphocytic	A tumor-like mass with edema	The left cerebellum	Corticosteroids	Recovered well without residual deficits on 3 month follow up

The present patient had a single, tumor-like enhancing lesion associated with extensive vasogenic edema on MR imaging. Conversely, PACNS presenting multiple lesions that also mimic aggressive multifocal cerebral neoplasm have been reported (26, 31–34). A retrospective study of 10 tumor-like PACNS cases in France found that the number of patients with single and multiple lesions was similar (4 and 3 patients, respectively) (35). The Mayo Clinic analysis showed that nearly 83% of the multiple lesions tended to be bilateral (7). Panchal et al. (33) and Michael et al. (36) reported cases in which all of the multiple lesions were confined to one cerebral hemisphere, while the other cerebral hemisphere, cerebellum, and brain stem were completely normal. After the initiation of appropriate therapy, both patients had rapid improvement clinically and on MR imaging.

Studies of the vessel lumen include digital subtraction cerebral angiography (DAS), MRA, computed tomography angiography (CTA), and transcranial doppler (TCD). The sensitivity of these methods is not high, but positive results can be used as evidence for suspected cases of PACNS. When the vessel diameter is <0.2 mm, DSA, currently the superior imaging technique, is often negative. When large blood vessels are involved, especially if the changes are present bilaterally and in multiple arteries, they often show alternating segments of stenosis, referred to as beading, and normal or dilated segments and arterial occlusions (8, 37, 38).

Differentiation between tumor-like PACNS lesions and actual CNS tumors is challenging because of similar MR patterns. The diagnosis is essential for medical intervention strategies because the treatment of these two types of diseases is completely different. If untreated, patients with PACNS will deteriorate rapidly. Early recognition and therapy may help improve outcomes in patients with PACNS. However, the current diagnostic methods lack specificity, and some seldom-used methods may help clinicians distinguish them.

MR spectroscopy may be very beneficial for the evaluation of patients presenting with tumefactive lesions of the CNS. Typical findings in glioma include high choline, low N-acetyl-aspartate (NAA) peaks, and high lactate levels (23). Elevated glutamate and/or glutamine peaks were observed in three case reports, the local accumulation of which was due to the breakdown of both neural and glial elements and the associated adjacent astrocytic response under inflammatory conditions (25, 33, 39). Elevation of these peaks may suggest an inflammatory etiology even if the imaging pattern is more consistent with a neoplastic disorder. Researchers also observed elevated choline/NAA ratios and lipid/lactate peaks to varying degrees. Nonspecific findings were observed in the degree of choline, NAA peaks, and lactate levels (20, 26, 33, 39). Furthermore, the fractional anisotropy (FA) values on DT imaging in a case reported in 2007 were very low in both the peripheral enhancing region and the central necrotic region, which tend to be high for tumors classified as high-grade gliomas (40, 41). This finding enabled us to eliminate a glioblastoma diagnosis and may develop into a new method of identification of PACNS after further study in a limited group of patients (39). Some cases also revealed changes in perfusion MR imaging and CT, even so other studies also described their potential usefulness for diagnosis of vasculitis. When conventional MR imaging and angiography are normal, perfusion imaging may provide information related to focal perfusion abnormality (25, 26).

Advances in imaging such as high resolution magnetic resonance imaging (HR-MRI) provide information regarding the vessel lumen and vessel walls, particularly the wall structure of the intracranial blood vessels, which cannot be obtained by luminal imaging (16, 42). Thickening of large vessels and extramural enhancement revealed by HR-MRI may be more specific for the diagnosis of PACNS (43) that is not always seen in neoplastic disorders of the CNS. Although to define specificity and the role of HR-MRI in the diagnostic process, further studies of inflammatory and non-inflammatory vascular disorders are needed (6), HR-MRI can also be used to distinguish tumor-like lesion PACNS and high-grade glioma.

To date, brain biopsy remains the gold standard for the diagnosis of PACNS. Special stains and cultures of pathologic samples are essential to confirm that the lesions do not represent a secondary form of vasculitis. Immunohistochemistry and molecular analysis of T-cell and B-cell clonality should also be considered to exclude CNS lymphoma, a common mimic of PACNS (6, 15). Histological findings in PACNS commonly include a lymphocytic inflammatory reaction with variable numbers of plasma cells, histiocytes, neutrophils, and eosinophils (11). A study showed that 89% of PACNS patients with tumor-like lesions presented lymphocytic patterns, similar to other PACNS subtypes. Granulomatous patterns were next, accounting for approximately 14% (35). Unfortunately, immunohistochemistry and molecular analysis of T-cell and B-cell clonality was not completed in our patient because our neurosurgeons did not doubt the diagnosis of the patient's glioma before surgery. Although glucocorticoid therapy was effective cannot exclude CNS lymphoma, hypermetabolic lesions of the CNS were not found in FDG-PET (44), so CNS lymphoma can also be excluded. Tumefactive demylanting lesions (TDLs), another common mimic of PACNS also need pathologic examination to distinguish. For our patient, according to initial the MRI appearance, TDLs should be considered. It is histological examination that help us to make a clear diagnosis (45).

Of note, after the initiation of therapy, there was a rapid improvement both clinically and on MR imaging in the present case. However, an analysis revealed that for tumor-like PACNS patients, outcomes were favorable in cases involving a combination of glucocorticoids and cyclophosphamide compared to those treated with glucocorticoids alone (35).

## Conclusions

Tumor-like PACNS cases are rare. Such patients are often misdiagnosed with high-grade gliomas because of T1-weighted low signal intensity, T2-weighted high signal intensity, gadolinium enhancement, and significant edema and mass effect. Preoperative radiological evaluation may not always directly provide the exact diagnosis. Brain biopsy is the gold standard diagnosis, but this is an invasive test accompanied with a small but significant risk of serious complications. Imaging procedures may be a key part of the workup of patients with PACNS. Some uncommonly used methods such as MR spectroscopy may help clinicians distinguish PACNS from its mimics. Furthermore, early combined therapy with aggressive immunosuppressants and steroids may help improve outcomes in PACNS patients.

## Ethics Statement

This study was carried out in accordance with the recommendations of the Human and Research Ethics committees of the First Hospital of Jilin University with written informed consent from the patient. The patient gave written informed consent in accordance with the Declaration of Helsinki. Written informed consent was obtained from the patient for the publication of this case report. The protocol was approved by the Human and Research Ethics committees of the First Hospital of Jilin University.

## Author Contributions

HJ, YQ, and F-LZ: drafted the manuscript. YQ: acquisition of data. HJ, Z-NG, G-ZC, and F-LZ: analysis or interpretation of data. YY: study concept and design.

### Conflict of Interest

The authors declare that the research was conducted in the absence of any commercial or financial relationships that could be construed as a potential conflict of interest.
